# Considerations for mRNA Product Development, Regulation and Deployment Across the Lifecycle

**DOI:** 10.3390/vaccines13050473

**Published:** 2025-04-28

**Authors:** John H Skerritt

**Affiliations:** Faculty of Medicine, Dentistry and Health Sciences, University of Melbourne, Melbourne, VIC 3010, Australia; john.h.skerritt@gmail.com or john.skerritt@unimelb.edu.au

**Keywords:** mRNA, vaccine development, regulation, safety, lifecycle, platform technology

## Abstract

With the successful deployment of several mRNA vaccines against SARS-CoV-2, an mRNA vaccine against RSV (respiratory syncytial virus) and a large pipeline of mRNA products against other infectious diseases, cancers and rare diseases, it is important to examine the whole product lifecycle. mRNA technology enables product design, testing and manufacturing systems to be rapidly developed, but these advantages can be lost if other factors that determine public access are not closely considered. This review analyzes key aspects of the mRNA product lifecycle including candidate design, manufacturing, quality systems and product safety and storage. Regulatory thinking is well advanced in some countries but not others, but more thought on the regulation of mRNA vaccines outside of a pandemic situation as well as mRNA therapeutics including individual neoantigen therapies and rare disease treatments is needed. Consumer acceptance—the “social license to operate” around mRNA products—is critical for their uptake, particularly outside of a pandemic.

## 1. Introduction

The development of mRNA vaccines and therapeutics has demonstrated a number of advantages in product research and development and in the consistency of development processes and production that are readily scaled to suit the size of the patient cohort and product demand. The technology has proven broadly applicable to include both single protein products (e.g., as in monovalent COVID-19 vaccines or single enzyme replacement therapies), complex proteins (e.g., multiple sequences in CMV vaccines or individual neoantigen oncology therapies) or combinations of proteins (e.g., in seasonal influenza—COVID-19 vaccines). By utilizing antigens in products as different as a vaccine for RSV or an individualized neoantigen therapy for melanoma, mRNA technology has demonstrated wide applicability and a high chance of technical success. Similarly, for monogenic rare diseases, identification of the mechanism of the disease where it can be attributed to a missing or malfunctioning enzyme or other protein in a metabolic pathway also enables the targeting of mRNA therapeutics.

The application of platform technology [1] has also facilitated product research and development and manufacture, and promises to streamline regulatory review. There are a range of definitions of platform technology used internationally, and the concept is applied to a wider range of products outside mRNA vaccines and therapeutics. For example, the US Food Drug and Cosmetic Act defines platform technology as “a well-understood and reproducible technology, which may include a nucleic acid sequence, molecular structure, mechanism of action, delivery method, vector or a combination of any such technologies that FDA determines to be appropriate, where the sponsor demonstrates that the technology: is incorporated in or used by a drug/biological product and is essential to the structure or function of such drug/biological; can be adapted/incorporated/used by more than one drug or biological product sharing common structural elements; (and) facilitates the manufacture or development of more than one drug/biological product through a standardized … process”.

The development of mRNA vaccines and therapeutics is very similar for very different diseases and conditions, with a similar mRNA sequence backbone and the same lipid nanoparticle technology utilized in different products within a therapeutic area, and the only significant change being in the antigen-encoding mRNA sequence. This enables the experience with manufacturing, quality controls/analytical testing and preclinical testing (such as biodistribution and toxicology studies) to be adapted for other products [1]. Cell-free manufacturing of the mRNA sequence from a DNA template, the microgram doses of mRNA products (rather than gram-scale doses required for many monoclonal antibody therapeutics), and similarities in manufacturing processes between mRNA products for very different indications provide for fast, flexible and well-controlled manufacturing. mRNA manufacturing at a comparably modest physical scale has enabled the manufacture of billions of doses of COVID-19 vaccines and enables a move away from product-dedicated pharmaceutical and vaccine manufacturing plants that were a model for the pharma industry for some decades.

The focus of this review is on mRNA product development, regulation and deployment across the lifecycle. The author is aware that the term “lifecycle” as applied to pharmaceuticals and vaccines can mean different things for mRNA product manufacturing [2], regulation [3], commercialization or supply chain. The review is not a meta-analysis or systematic review of all of the available literature but rather is a narrative review focusing on the most relevant aspects of the topic of “mRNA product development, regulation and deployment across the lifecycle”.

## 2. Considerations for mRNA Product Design—The Beginning of the Lifecycle

In this review, I take a broader view of the term “lifecycle”, and highlight some areas that have not been sufficiently explored with respect to mRNA vaccines and therapeutics. In particular, there is a focus on the critical aspect of mRNA product development that involves conceptualization through to population or patient use. Aspects of the design of mRNA products are reviewed, including some of the advances in mRNA design that have been enabled by the use of AI technologies. Together with coauthors, I earlier reviewed aspects of the development, manufacturing and regulatory lifecycle, particularly as they relate to preclinical and clinical development, manufacturing steps, quality assurance and platform technology aspects [1].

A range of considerations specific for the lifecycles of mRNA vaccines and therapeutics, and the regulatory challenges these entail, are reviewed in this paper. Clinical trials are also a critical part of the mRNA product development lifecycle. While the platform approach can guide clinical development, with the exception of variants to existing vaccines, new product-specific clinical data are usually required for each product. As a comparatively new commercial technology, it is important that safety and consumer/patient considerations are also discussed.

This review focuses on vaccines and therapeutics developed from conventional mRNA and unmodified lipid nanoparticles. However, it is recognized that other “sub-platforms” involving self-amplifying mRNA (sa-mRNA, [4]), circular RNA [5], other lipid nanoparticle (LNP) variants [6] and other mRNA technologies, involving delivery through other nanoparticles, dendritic cells or CAR-T cells, are also among the other therapeutic technologies using mRNA [7,8].

Several other reviews [4,9,10] have described processes of optimization of mRNA sequences and structure for use in vaccines and other products, so they will only be summarized briefly here. mRNA sequences utilized in vaccines and therapeutics have five main structural elements: a 5′ cap which influences stability and translation efficiency; the 5′ untranslated region which acts as a binding site for ribosomes to initiate translation; the coding region that contains the sequence for the protein to be translated from the mRNA; a 3′ untranslated region that also affects mRNA transport, stability and translation; and a poly-adenosine (A) tail, which protects the mRNA from degradation.

In addition, modification, deletion or insertions of particular nucleosides in the mRNA sequence can be pursued to reduce stimulation of the innate immune system by RNA and to increase translation of the encoded protein sequence [11]. Kariko and colleagues [12] made the critical discovery that chemical modification of nucleosides could down-regulate RNA-mediated innate immune activation and reduce stimulation of pattern recognition receptors such as the toll-like receptors 3, 7 and 8, which can lead to interferon alpha and gamma production as well as interleukin 12 synthesis. In doing so, unwanted immunogenicity and reactogenicity can be decreased. Incorporation of pseudo-uridine into the mRNA sequence increases translation while avoiding recognition by pattern recognition receptors or RNA-dependent protein kinase activation, which can lead to RNA degradation through stimulation of nuclease production [13].

Some differences in mRNA sequence and structure are required for vaccines and therapeutics [14]. Prophylactic vaccines for infectious diseases require products that can ideally stimulate both B- and T-cell immunity, while mRNA oncology therapeutics are required to stimulate a significant cytotoxic cellular immune response so that cancer cells are eradicated. This can make antigen/epitope design for mRNA cancer therapeutics quite challenging, as T-cell receptor epitopes are generally less well understood [15]. In contrast, while mRNA vaccines and mRNA oncology therapeutics are assisted by the immunogenicity of the mRNA itself, in mRNAs for enzyme/protein replacement in rare diseases, activation of the innate immune system by the mRNA therapeutic is quite undesirable. This is reduced in these products through nucleoside modification to increase the durability of expression of the mRNA [16], although recent studies have shown that optimizing or attenuating ribosome translational load along with optimizing codons leads to the greatest mRNA stability [17,18]. Some software packages have been designed to optimize the design of therapeutic mRNAs [16]. While these context-specific considerations exist for the selection of the target protein to be expressed by the mRNA and in the design of the mRNA, common manufacturing elements, such as raw materials, unit operations or optimized process parameter setpoints, may be sharable in a platform-based approach even between mRNAs intended for highly diverse therapeutic applications.

### Applications of Artificial Intelligence (AI) and Machine Learning

The power of mRNA vaccine and therapeutic technologies also brings with it complexity. The ability to potentially design and translate an essentially unlimited number of amino acid variants in a protein sequence, the requirement to determine the most effective codon choices for the amino acids in the protein sequence expressed by the mRNA and the need to decide on particular codon modifications mean that there are many thousands to millions of potentially feasible alternatives for each candidate mRNA. For example, due to the existence of synonymous codons alone, there are around 2.4  ×  10,632 candidate mRNA sequences for the codons encoding the 1273 amino acid SARS-CoV-2 spike protein [19]. Optimization of the untranslated mRNA region can also be undertaken using AI and machine learning approaches [20].

While the goal of exploring potential alternatives within mRNA sequences is clear—so as to increase the stability and expression of the mRNA and improved commercial manufacturability—it is not feasible to assess many of these alternatives through laboratory experimentation. An increasing number of research groups have reported the use of computational, bioinformatic and AI approaches for mRNA vaccine design [19,21,22,23,24,25], with commercial mRNA vaccine developers also extensively utilizing these approaches for mRNA vaccine and therapeutic design.

Computational methods, incorporating AI and machine learning have been used to optimize mRNA sequences and structures in several ways. These include the design of untranslated regions to enhance ribosome loading and translation efficiency; optimizing codon usage in the open reading frame to increase translation efficiency; stabilization of the overall sequence and avoiding specific sequence motifs that could lead to instability or poor expression [26,27]. These approaches were applied to the design of vaccines for SARS-CoV-2 [28,29] and are a critical part of successful mRNA cancer vaccine development [30,31]. Imani et al. [31] review a range of bioinformatic tools, some of which incorporate machine learning approaches for potential utility in mRNA cancer vaccine epitope prediction, codon optimization, secondary structure prediction, protein structure prediction and in the design of mRNA-LNPs. Some tools incorporate AI.

Added to these approaches are other modifications that can be investigated using AI. Several AI applications aim to reduce the toxicity or immunogenicity of sequences and their expressed proteins and potentially optimize the biodistribution of the protein translated from the mRNA. Machine learning models are particularly useful in predicting immune responses from the protein sequence to be encoded by the mRNA open reading frame and thus predict epitopes in mRNA vaccines. The use of AI and machine learning approaches reduces unwanted immune responses, e.g., from mRNA therapeutics that are typically to be dosed repeatedly. Machine learning can quantify both expected B-cell [32] and T-cell [33] responses. An alternative approach uses deep learning through artificial neural networks that identify complex patterns in protein sequences and can, for example, predict peptide affinities for major histocompatibility molecules [34].

AI approaches can therefore enhance several steps in the design and development of mRNA vaccines and therapeutics [22,25]. Where there are several potential protein antigen candidates for a vaccine, these can be ranked through AI and their immunogenicity can be predicted. Deep learning approaches supported by artificial neural networks can assess large databases of proteomic and genomic sequences to identify vaccine candidates, especially when a single dominant epitope has not been identified in earlier studies. This approach has particularly been applied to the design of mRNA vaccine candidates for infectious disease [20]. Potentially undesirable cross-reactions of vaccines with human proteins can also be predicted. Computational approaches to predict immunogenicity predate the application of AI but the methods now used with mRNA vaccines enable much more extensive and rapid investigation, and some newer AI models simulate both humoral and cellular immunogenicity.

A “LinearDesign AI tool” that increased mRNA sequence stability through the creation of folded structures [19] resulted in significantly higher antibody responses in mice with candidate COVID-19 and varicella-zoster vaccines. Structural reconfiguration of the mRNA molecules was conducted by algorithmically determining the most stable configurations of nucleotide sequences, allowing the mRNA to loop back on itself and form intramolecular double-stranded structures. AI approaches have also been used to predict suitable epitopes in mRNA cancer vaccines, for example, predicting the MHC presentation ability and immunogenicity of neoantigens [35,36] including in the design of individualized neoantigen therapeutics and the design of mRNA sequences and structures that optimize antigen presentation [37]. The identification of suitable LNP formulations (particularly the ionizable lipid component/s) is still largely conducted empirically through laboratory screening of candidates. However, as the research and patent literature on LNP formulations increases, recently, several groups have published machine learning approaches that interrogate this data source [38] and potentially to identify new ionizable lipid structures for use in LNPs [39] or to use machine learning to optimize the composition of LNPs [40].

## 3. Clinical Trials of mRNA Products

An increasing number of mRNA products are moving from preclinical development into clinical trials; we [1] have recently reviewed those in late-stage (phase 2 or 3) trials. mRNA products are highly amenable to the application of platform technology approaches for their preclinical development, manufacturing, controls and analytical methods, but clinical trials are typically required for each new product. There is the potential to use clinical trial results from related platform vaccines to determine possible doses, but only as a guide. Some conclusions about reactogenicity and safety based on impurities or concentrations of contaminants may also be possible. Exceptions to requiring full clinical data packages are mRNA vaccine to viral variants (e.g., for SARS-CoV-2), and it is expected that as mRNA vaccines for seasonal influenza become available, annual strain updates should also be able to be made without a regulatory requirement for clinical data [1]. Preclinical data may be able to support a related platform product, such as pharmacokinetic and biodistribution studies, toxicology data as well as assays for, and information on, potential immune responses and reactogenicity. Some vaccine-specific data will still be needed.

The requirements for clinical trials for mRNA products are in general no different than for other drugs, although as for other vaccines, the numbers of participants enrolled in vaccine trials are typically somewhat larger than for therapeutics. Information from products with similar mRNA sequence design principles and secondary structures, and LNP nature and composition can also provide useful platform information in summarizing preclinical development data.

While several billion doses of mRNA vaccines have been administered globally, there will still be individuals who may have concerns about taking part in a clinical trial of an mRNA product. The clinical trial informed consent information therefore should make it clear that the product in the active arm of the clinical trial is an mRNA product. Reactogenicity is a common but typically mild and transient adverse event for mRNA COVID-19 vaccines, so it is recommended that trial participants are advised about this possibility. In addition, while advising trial participants of the importance of seeking urgent assistance for potentially serious adverse events, it is also important not to confuse common reactogenicity symptoms with more serious adverse events.

The COVID-19 pandemic demonstrated that highly effective mRNA vaccines could be developed in a matter of months against a lethal viral pathogen of global concern. With 13.7 billion doses of COVID-19 vaccines administered as of August 2024, a significant majority of them being mRNA vaccines, the SARS-CoV-2 pandemic provides a real-world demonstration of vaccine safety and efficacy [41]. This experience can be utilized for other emerging diseases, and the ability of the technology to serve as a platform was amply demonstrated, meaning that now, and in the future, neither developers nor regulators will be required to start “from first principles” to develop new mRNA vaccines for other pathogens. Certain elements of mRNA medicines, such as mRNA design, LNP composition and manufacturing processes and controls, which were established through the development of mRNA vaccines in the COVID-19 pandemic, should be applied by developers, where appropriate, to deepen the evidence of product and process understanding provided in applications for new mRNA medicines. Similarly, this understanding can be employed by regulators to simultaneously provide additional rigor to their assessment and increase the efficiency of their review for elements which are fixed between products.

### Challenges with Emerging and Tropical Diseases

mRNA as a platform technology can accelerate the development of vaccines for emerging and tropical diseases [42]. While highlighting the benefits that mRNA platform technology provides in rapid, scalable vaccine development, and the fact that the equipment used for mRNA-LNP products could be interchanged between products, including vaccines against different pathogens, the authors [42] described challenges for the development of vaccines against individual diseases. These include, for particular diseases, the lack of animal models, poorly established correlates of protection, clinical development challenged by sporadic and unpredictable outbreaks, variable antigenic diversity and challenges with antigen design and the requirement for containment facilities for preclinical challenge studies. Human challenge studies may also not be feasible or ethical in certain cases.

In cases when an emerging disease affects populations sporadically, clinical trials for the development of vaccines can pose challenges. The sporadic or low rates of infection could mean that prohibitively large populations would need to be enrolled to demonstrate clinical efficacy. In these cases, correlates of protection (an immune function that correlates with and may be biologically responsible for efficacy) or surrogates of protection (an immune marker that can be used to predict efficacy when the actual correlate of protection has not been firmly established) can be used in a smaller population of healthy subjects. However, establishing immune biomarkers as adequate surrogates of clinical benefit is a challenge in itself [43]. Understanding the elements of the immune response that are involved in protective mechanisms is critical to developing reliable biomarkers that can help expedite the development process. Novel immune profiling tools will enable a more extensive characterization of the protective mechanisms and help identify those biomarkers.

Expedited regulatory pathways such as the US FDA’s Accelerated Approval pathway provide an option for faster approval of medicines that address a significant public health need ahead of the demonstration of clinical efficacy, provided that a surrogate marker reasonably likely to predict protection is identified, and that a clinical study to confirm efficacy is planned or underway [44].

Several organizations are developing—or have already developed—vaccines for highly infectious diseases which also have a high mortality rate. While the clinical trial considerations for mRNA vaccines for these diseases do not necessarily differ from other types of vaccines, it is worth outlining some considerations that may be relevant to clinical trials of these vaccines. Some mRNA candidate vaccines include those against Nipah virus [45,46,47], Ebola disease [48], rabies [49], Zika [50], mpox [51] and mRNA-encoded Chikungunya virus monoclonal antibodies [52].

Global regulators and the World Health Organization (WHO) have considered alternate approaches when the usual expectation of the completion of phase 2/3 human trials of a product may be neither practical nor ethical, although demonstration of some form of clinical benefit in a randomized controlled interventional study remains the gold standard. The US FDA “animal rule” has been in place for over 20 years [53]. Related to this is the animal model qualification program, which provides guidance on appropriate drug development tools, including animal biomarkers, and how they can potentially be reviewed and formally qualified by US FDA [54]. For a product to be eligible under the animal rule, the mechanism of action of the disease needs to be understood reasonably well. Animal studies may be required in more than one species and must be closely related to the desired benefit (protection/immunity in humans), and data on the drug or vaccine pharmacokinetics and pharmacodynamics in animals and humans enable the selection of an effective human dose. However, many viruses have very tight host species specificity and animal challenge models are not available for a human pathogen, creating difficulties for the interpretation of animal studies with related animal host-specific agents. There are also post-market requirements that FDA imposes for drugs and biologicals approved under the animal rule, in particular, the need for studies in humans if/when there is a disease outbreak.

The European Medicines Agency (EMA) also developed guidance around the animal rule [55]. There are some important policy differences to the US FDA, with a greater focus on demonstrating safety and having strong pharmacovigilance systems in the presence of unclear efficacy. The USFDA guidance differs from the EMA guidance in that it goes into some detail in prescribing animal study requirements. It also requires information to demonstrate why human studies are not feasible. These may include reasons why extensive human clinical studies could not be conducted, such as low infection incidence outside of an epidemic or pandemic situation. Ethical reasons may also be factors—the use of an unvaccinated control group when a disease with high mortality is circulating could be seen as unethical. Regional regulatory reliance involving leading reference agencies will also be important in supporting rapid regulatory review and deployment of mRNA and other vaccines against epidemic diseases of local importance [56].

## 4. CMC Lifecycle Considerations in mRNA Product Manufacturing and Deployment

### 4.1. Manufacturing and Supply Chain Considerations

Manufacturing scaleup of mRNA-LNP vaccines had to be rapidly addressed during the SARS-CoV-2 pandemic. Even for companies already involved in mRNA-LNP manufacturing under Good Manufacturing Practice (GMP) conditions, within a few months, manufacturing had to transition from the milligram to sub-gram quantities required for preclinical and early-stage clinical development to being able to manufacture and deploy millions to billions of doses globally. There are some advantages of mRNA-LNP vaccine manufacture compared with alternate production technologies. The culture volumes are typically much lower and processes do not require the use of eukaryotic (including mammalian) cells, which reduces the risk of contamination with adventitious viruses. Also, a very similar (platform) approach is used for the manufacture of most target antigens.

However, the sudden increase in scale of manufacturing did lead to challenges in sourcing sufficient quantities of pharmaceutical grade raw materials [57]. The components of the in vitro transcription process that produces the mRNA, including enzymes used in capping steps, must be obtained from certified suppliers that guarantee that all the material is animal component-free and GMP-grade [58]. Several of the critical raw materials were not included in major pharmacopeia. The removal of product-related impurities (e.g., enzymes, residual nucleoside triphosphates and DNA template, and aberrant mRNAs) following mRNA synthesis is crucial as they can lower subsequent translation efficiency and cause unwanted immune stimulation. Chromatographic and other methods that were able to perform at sufficient scale to purify mRNA products also required rapid development [59].

Similar manufacturing and scaleup challenges have been identified for LNPs. However, these were successfully addressed during the COVID-19 pandemic, despite the manufacture of the proprietary lipids that are used in LNPs being a specialized process, with few commercial-scale suppliers [60]. Several tons of lipid were required annually for manufacturing LNP for COVID-19 vaccines during the height of the pandemic [61]. The commercial-scale combination of mRNA and LNP to produce mRNA-LNP vaccines can also be challenging. Mendonca et al. [62] have summarized the advantages and disadvantages of different approaches to combining mRNA and LNP, including T-junction mixing, hydrodynamic flow focusing and the use of a staggered herringbone and toroidal mixer. They emphasize the importance of conducting stability studies on the mRNA-LNP and the use of stabilizers such as buffers, surfactants and cryoprotectants.

Assays used for quality control also needed to be validated. Some were already well established, such as DNA content, double stranded RNA, bioburden and endotoxin, but required adaptation, including analysis of LNP-encapsulated mRNA [63]. Other tests such as in vitro potency tests and tests that could distinguish levels of components in bivalent vaccines required rapid development by manufacturers and validation by regulatory lot release laboratories. Warne et al. [64] have summarized the critical challenges that Pfizer had to overcome in delivering three billion doses of the SARS-CoV-2 mRNA vaccine in the first year of its rollout, emphasizing that the in vitro transcription reaction and LNP had previously only been conducted at laboratory and pilot clinical scales. The authors listed the four critical challenges as follows: securing critical resources to reduce supply chain uncertainty; ensuring the high quality of vaccines made at different scales and sites; designing dose forms for convenience and transporting vaccines to people around the world. On the latter, one of the key innovations was the development of a dry-ice pallet shipper containing a GPS tracker, following extensive shipping simulation studies.

### 4.2. Increasing the Stability of mRNA-LNP Vaccines and Therapeutics

With the need for rapid development and deployment of mRNA vaccines during the COVID-19 pandemic, manufacturers initially took a conservative approach to storage and shipping conditions for these products [65]. This was because it was well recognized that in aqueous solution, mRNA is inherently unstable, as both its single-stranded nature and the presence of a 2′ hydroxyl group can enable hydrolysis of neighboring phosphodiester bonds, resulting in cleavage of the mRNA. Longer mRNA sequences can be less stable [66]. Several approaches can be used to stabilize mRNA prior to encapsulation in an LNP, including the use of buffers and sucrose [67,68]. The design and modification of the mRNA sequence and its secondary structure are also important in increasing its stability, including nucleotide selection, modification of the 5′ cap and elongation of the poly-A tail [69,70]. LNP composition also directly affects the storage stability of the mRNA as the mRNA forms a complex with ionized lipids.

Encapsulation can protect mRNA from enzymatic degradation, but interactions with LNP components have also been reported to increase degradation risks. Drug product formulation processes can also affect stability. Changing the buffer composition of the latter product from a phosphate buffer to a Tris buffer increased the shelf life of the defrosted product [70].

The deployment of COVID-19 mRNA vaccines in some developing countries has been challenging, given the storage and shipping requirements [71]. As a result, a range of other approaches, such as the development of lyophilized candidate vaccines, has been investigated. However, lyophilization does have the disadvantage that the presentation of the product is more complex (e.g., two vials rather than a pre-filled syringe) and extra steps, potentially introducing vaccinator errors, can occur in the reconstitution steps.

The stability of mRNA-LNPs was increased by lyophilization [72,73], with no loss in efficacy after 3–6 months at room temperature; although, some loss of efficacy has been seen in other studies [74]. An Indian lyophilized mRNA COVID-19 vaccine, GEMCOVAC-19, has received regulatory approval in that country. In GEMCO-VAC-19, the mRNA is attached to the surface of a nano-emulsion, rather than being encapsulated in LNPs (https://gemcovac.com/). The first self-amplifying mRNA COVID-19 vaccine to obtain regulatory approval (Arcturus ARCT-154) was also a lyophilized product. Sanofi’s current mRNA seasonal influenza candidate vaccine (SP0237) is also provided lyophilized, as is Moderna’s cytomegalovirus candidate vaccine (mRNA-1647 [75]). However, lyophilization does not automatically increase the stability of mRNA-LNP products. The typically low concentration of mRNA in the products often requires the use of cryoprotectants and/or bulking agents, and careful reconstitution is also required to avoid the appearance of aggregates and particulate matter in the vaccine prior to administration [76,77,78].

While, when successful, the long-term stability of lyophilized vaccines enables their manufacture and stockpiling ahead of an outbreak or regional epidemic [79], there are some disadvantages to lyophilization. Freeze-drying adds to manufacturing costs, while reconstitution of lyophilized vaccines can introduce particulates or aggregates and add to product handling and the risk of errors. To increase convenience, and avoid dosing errors, a prefilled syringe presentation can be developed. While syringes are shipped and supplied frozen, they are a convenient dose form, as they can be stored thawed in refrigerated conditions.

Another way of increasing the stability of mRNA vaccines is by designing shorter antigens. While the ancestral mRNA-1273 vaccine encodes the full-length SARS-CoV-2 Spike (S) protein, mRNA-1283, a next-generation vaccine intended to prevent disease caused by SARS-CoV-2, encodes only two regions of the S-protein which contain major viral neutralization epitopes, together with a linker and a membrane anchor sequence. The shorter mRNA length of mRNA-1283 facilitates higher levels of protein expression and enables longer storage at refrigerator temperatures [80]. Short, chemically synthesized mRNAs neoantigen vaccine formulations also demonstrated high levels of stability compared with in vitro transcribed mRNA [81]. A further approach is to provide mRNA vaccines through the use of micro-needle array patches [82,83,84]. In these devices, which also avoid the needles and syringes traditionally used for vaccinations, the mRNA-LNP vaccine is typically either air dried onto the micro-needled or included in dissolvable polymeric matrices. The devices can be stored at refrigerator or ambient temperature, and the vaccine’s thermostability makes the presentation suitable for use in low- and middle-income countries. Both the Gates Foundation and CEPI (Coalition for Epidemic Preparedness Innovations) have invested in clinical trials for mRNA vaccine patches.

Finally, modifications to the carrier for mRNA can also provide significant improvements in stability. Gerhardt et al. [85] developed a thermostable lyophilizable nanostructured lipid carrier for mRNA vaccines, others [86] have developed a biodegradable polyethyleneimine-coated porous silica nanoparticles-based mRNA delivery platform and several studies have used hybrid nanoparticles with an inorganic core and an organic shell [87].

## 5. Regulatory, Lifecycle and Deployment Considerations of mRNA Therapeutics

### 5.1. Rare Genetic Diseases

The development of biomarkers or genetic tests that can identify relevant patient populations suffering from rare metabolic diseases establishes a path for drug development. Several of the newer approaches to the treatment of these rare diseases, including mRNA therapies, address the root causes of the disease, e.g., through the replacement of a deficient or mutated enzyme. Development is not straightforward though as the understanding of the epidemiology and natural history of many rare diseases can be quite poor [88].

Gene therapies for rare diseases focus on permanently modifying the defective gene. These approaches have had a longer history than mRNA therapies, which are currently at the clinical trial stage. More than 10 gene therapies for non-cancer rare diseases have been approved by the US FDA. However, there are several limitations to the widespread use of these medicines. The gene therapy products have long manufacturing cycles and because they can require arduous preconditioning with chemotherapy drugs and in-or-outpatient infusions, they may not be well tolerated by patients. Other potential limitations include the inability to reverse gene therapies, risk of incorporation of the target gene into the nucleus and immunity to some viral vectors. Where they exist, treatment alternatives that can also help manage conditions such as hemophilia and may be preferred by affected patients.

mRNA treatments consist of an LNP-encapsulated mRNA encoding for the functional version of the defective protein, and therefore only produce transiently expressed proteins and do not alter the patient’s nuclear DNA. These treatments offer an efficient alternative to gene therapy for those patients that may not be eligible for gene therapy or for which there is no treatment option. mRNA therapeutics are anticipated to have applicability to treat rare metabolic and other diseases, particularly those that are caused by a single gene (monogenic) and where the protein expressed by the defective gene requires restoration or replacement [89,90]. A feature of proteins encoded by therapeutic mRNAs is that they can be expressed in targeted organs, using features such as the composition of the lipid nanoparticles, and the route of administration, and in specific cellular compartments—secreted, transmembrane, within the cytosol or mitochondria—as determined by the signal peptide encoded by the mRNA.

A number of successful animal models have expressed exogenous mRNA from mRNA-LNP administration to treat three rare metabolic diseases: methylmalonic acidemia (targeting methylmalonyl-CoA [91,92,93], acute intermittent porphyria (targeting porphobilinogen deaminase [94]) and Fabry disease (targeting alpha-galactosidase A [91]). Other targets studied at the preclinical phase include hemophilia B (Factor IX), ornithine transcarbamylase deficiency [95], propionic acidemia [96], glycogen storage disease types 1a (glucose-6-phosphatase) and 3 (glycogen debranching enzyme), phenylketonuria (phenylalanine hydrolase), cystic fibrosis (transmembrane conductance regulator), maple syrup urine diseases (branched-chain alpha-keto acid dehydrogenase, BCKDH) dysfunction and arginosuccinyl aciduria (arginase) [97,98,99]. Several candidates have now moved into clinical trials [1], including for propionic acidemia [100], methyl malonic acidemia, cystic fibrosis, ornithine transcarbamylase deficiency, phenylketonuria, propionic aciduria and primary ciliary dyskinesia.

### 5.2. New Approaches Will Be Needed to Keep Pace with Therapeutics Development for the Large Number of Rare Diseases

There are about 10,000 rare diseases identified [101] but only about five percent of rare diseases or conditions have US FDA-approved products available [102]. Using the current “one-disease, one-product-at a time” regulatory approach, it would take hundreds of years for therapeutics for each of the currently known rare diseases to be developed and to receive regulatory approval.

Several approaches could improve the efficiency of the development and regulatory review of new therapies for rare diseases. These include approaches such as basket clinical trials (designed to evaluate a single treatment intervention for multiple diseases that share a common molecular alteration) [103] or “N of 1” trials, which involve detailed longitudinal studies and the use of historical controls [104,105]. One approach with great potential to improve development and regulatory licensure is the more systematic, standardized and controlled leverage of platform technology frameworks. It has been estimated that about 6500 rare diseases are caused by mutations in single genes [106] and these provide a readily addressable opportunity for new drug development using mRNA [107,108]. The platform technology approach has also been applied to the development of gene therapies using a disease-agnostic approach and adeno-associated virus (AAV) vectors [109].

In addition, when there are several different but related genetic defects affecting a particular metabolic pathway, it may be possible to use the platform approach to further streamline evidence generation and regulatory review; an example is the rare diseases resulting from either propionyl-coenzyme A carboxylase or methyl malonyl coenzyme A mutase, which are successive enzymes in the same metabolic pathway and for which mRNA therapeutics are currently under clinical development [110]. As each of these therapies would consist of the same mRNA/LNP technology with only the encoded sequence being different, a potential model for accelerating the development and availability of these treatments would be to include several of these enzyme replacement treatments that belong to the same metabolic pathway under one marketing authorization.

### 5.3. Lifecycle Considerations for mRNA Rare Disease Therapies 

Several considerations for the development of mRNA therapeutics for rare genetic disorders in addition to those contemplated for vaccine development need to be addressed [110,111,112,113]. While the quality and manufacturing/CMC aspects of product development will often be largely similar to those for vaccines, the preclinical development program will not. With these therapies often requiring multiple doses given weeks apart during long periods, it is important to determine how durable the effects of the therapies are and thus the required frequency of repeat doses in clinical trials.

Studies on the biodistribution and accumulation of the relevant mRNA, translated protein (and its fragments) and LNP/LNP fragments are important, as are studies on the delivery to targeted organs. Most therapies are currently administered intravenously and circulate through the liver so are particularly useful when it is a liver enzyme that is in deficiency, but if distribution to other organs is required, alternate mRNA-LNP design strategies and/or administration routes may be necessary. Extensive preclinical safety studies are required, including on the assessment of inflammatory markers, liver function tests and on the potential development of antibodies to the protein expressed by the mRNA. Efficacy studies may be required in several animal species, especially if rodent models are of limited relevance to the particular rare human disease. In clinical studies, the duration of effect of the mRNA rare disease therapy will depend on the target protein and the organ system affected, and these will need to be established for each therapy individually. An understanding of the natural history of the disease should inform whether it is acceptable or desirable for the translated protein levels to drop back to baseline levels in the periods between treatments.

### 5.4. Oncology—Individualized Neoantigen Therapies (INTs)

Tumor-specific neoantigens result from genetic alterations in cancers; they are recognized as foreign by the immune system and so are not subject to immune tolerance. The advent of next-generation sequencing and bioinformatic technologies has enabled these neoantigens to be targets for personalized cancer immunotherapies. mRNA, peptide and viral vector vaccines based on neoantigens rather than other tumor-associated antigens have several potential advantages [85,86,87,88,114] and have been applied to a range of tumor types in preclinical models and in clinical trials. Neoantigens are exclusively expressed by tumor cells, so the resulting T-cell responses are tumor-specific and damage to healthy tissue is minimized. As neoantigens are derived from somatic mutations, T-cell central tolerance of self-epitopes should be absent and a potentially robust immune response to the tumor could be induced. Finally, vaccine-boosted neoantigen-specific T-cell responses could persist and could possibly provide long-term protection against disease recurrence.

A range of therapeutic approaches to the use of neoantigens in cancer vaccines have been developed [107], including adoptive cell therapies (T-cell receptor engineered T-cells, CAR-T), dendritic cell vaccines, bispecific antibodies, peptide-based neoantigen vaccines and nucleic acid (RNA and DNA based) vaccines. mRNA therapeutics, however, have certain advantages over other approaches, including the following:

(1) Simultaneous delivery of multiple tumor antigens, reducing the risk of resistances or antigen loss or change;
(2) Full-length antigens can be encoded if required, enabling multiple epitopes to be presented;
(3) Induction of a broad T-cell response;
(4) Manufacture is rapid and scalable compared with some of the other approaches.

**Figure 1:**
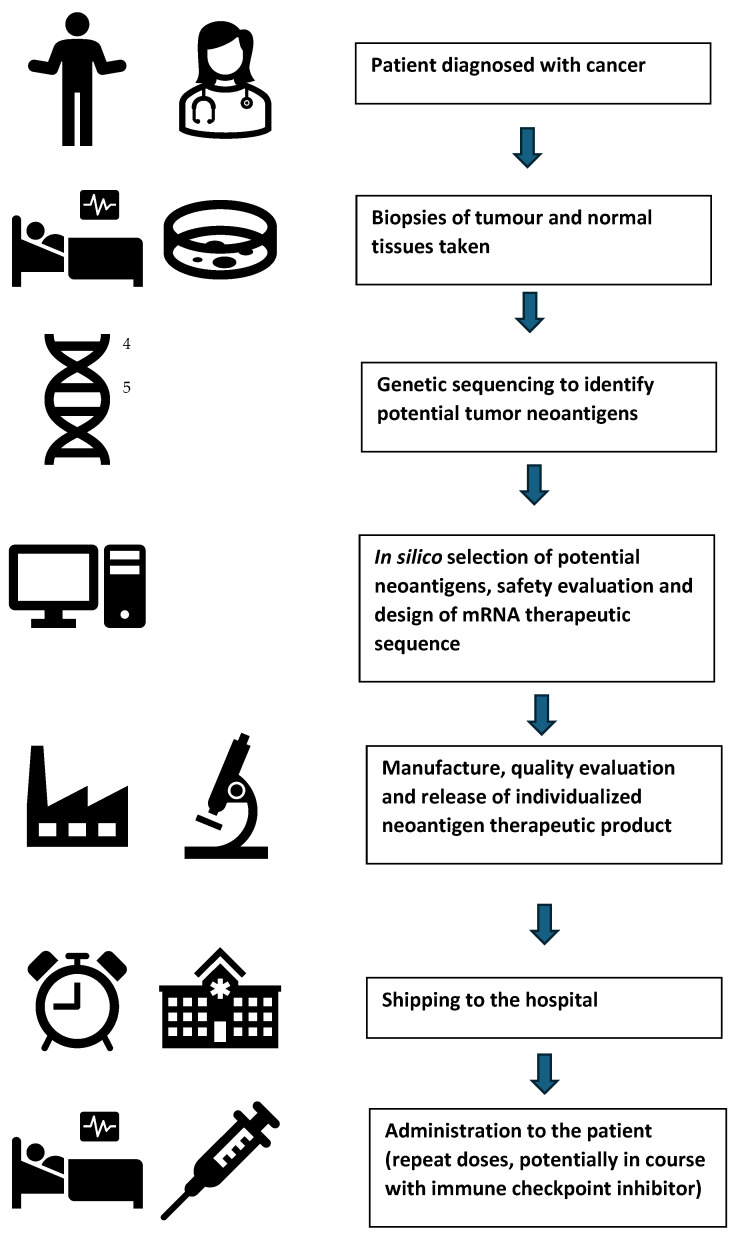
Development and administration of mRNA individual neoantigen therapies.

The development of mRNA individualized neoantigen therapies is summarized in Figure 1. The identification, prediction and validation of suitable immunogenic neoantigen sequences are a critical part of the mRNA individualized neoantigen therapy development. Whole exome sequencing, RNA sequencing and proteomic data can be used, but the identification of genome-expressed mutations and HLA typing of patients is required to best predict which neoantigens should be selected. Next-generation sequencing data of normal and tumor tissues from the same patient are compared so that (somatic) mutant peptide sequences are able to be identified. In combination with HLA typing information, HLA binding of particular sequences can be predicted in silico to ensure that there is strong T-cell recognition of as many of the potential neoantigens as possible.

Personalized cancer immunotherapies encoding individual tumor mutations are being trialed in melanoma, adjuvant non-small cell lung cancer, gastrointestinal cancers, other solid tumors, colorectal cancers, triple negative breast cancer and pancreatic cancer, with or without an immune checkpoint inhibitor or another oncology drug [108,109]. Applications of INT have continued to expand, specifically for applications in tumor heterogeneity and/or low antigen burden. This includes preclinical models in a range of glioblastoma and medulloblastoma brain tumors where selective gene capture and enrichment have been used to develop mRNA therapies [115]. INT is also continuing to address challenges including increasing the rates of identification of neoantigen peptide sequences that will bind MHC with high affinity and be reliably recognized by patient T-cells; loss of neoantigens as the cancer evolves; immunosuppressive tumor microenvironments; insufficient production of neoantigen-specific T-cells and limited neoantigen-reactive T-cell repertoire [116,117,118]. As the understanding of neoantigens increases, there is better identification of the most immunogenic ones using computational approaches [119,120].

mRNA technology is ideally suited to individualized neoantigen therapy as neoantigens can be identified and sequenced from an individual patient, and corresponding codon-optimized mRNA sequences can be produced and manufactured into an mRNA-LNP for that patient within weeks. The approach brings with it technical, logistical and regulatory challenges. First, suitable neoantigens may be difficult to identify in certain patient tumors, and/or it is difficult to predict the most immunogenic neoantigen.

In the phase 2 trial of an INT mRNA vaccine under development by Moderna and Merck, the minimal number of target epitopes per patient was 9, and 91% of patients received mRNA encoding the full 34 epitopes [119]. The product being trialed by BioNTech for pancreatic ductal adenocarcinoma (in combination with atezolizumab and several chemotherapy agents) typically contained up to 20 target epitopes per patient [111]. Other personalized neoantigen mRNA treatments are in clinical trials [121,122]—including a self-amplifying-mRNA booster for metastatic colorectal cancer (Gritstone Bio, Emeryville, CA, USA) and an mRNA treatment for advanced solid tumors (Stemira Therapeutics, Shanghai, China) [24,122]. However, the risk that the composition and predominance of different neoantigens evolve during the course of the disease and may change by the time the mRNA treatment is delivered to the patient remains an important consideration.

### 5.5. Regulatory Considerations for Individualized Neoantigen Therapies (INTs)

The manufacturing of large numbers of individualized vaccines will require simultaneous parallel end-to-end manufacturing at a very small scale for dozens or hundreds of versions of the product under Good Manufacturing Practice (GMP) conditions—effectively requiring “scale out”, rather than the typical “scale up” that occurs when a product is commercialized. Another consideration relevant for mRNA therapeutic development involves multi-dosing (e.g., nine doses of mRNA-4157 at three-weekly intervals). Therefore, more in-depth preclinical studies will be required, compared with mRNA vaccine development. On the other hand, compared to other oncology medicines, there is often a different approach to benefit–risk than for a prophylactic vaccine which is to be administered to large numbers of healthy adults and children.

It is anticipated that initial regulatory submissions for INTs will be made for potential approval against the particular cancer and disease paradigms that were the subject of the particular clinical trials (e.g., adjuvant or particular disease progression stages). However, having addressed the regulatory issues for the INT “platform technology within a platform technology”, development and regulatory review for similarly designed product against other indications could be facilitated. Because each product is unique for each patient to be treated, regulatory approaches will require adaptation to accommodate this.

Potential regulatory approaches to assess the manufacturing quality, and efficacy and safety of mRNA vaccines were discussed previously [1], noting that each individual neoantigen mRNA product is unique. Recently, Jonker et al. [123] reviewed the range of N-of-1 individualized therapies under development and noted that mRNA individual neoantigen therapies differ from other N-of-1 therapies in that they can be tested in clinical trials involving multiple patients with the same cancer type using the same endpoints. US FDA guidance [124] has indicated that each component of multiantigen vaccines may not be required to be individually evaluated, although it was emphasized that this would be on a case-by-case basis.

The South Korean regulator (Ministry of Food and Drug Safety, MFDS) has released regulatory guidance specifically on individualized neoantigen therapies [125]. The guidance is principles-based and emphasizes a focus on the assessment of controls of design and manufacturing processes for the INTs in potential regulatory review, although the guidance is not specific to mRNA products.

These principles include the following:

(1) Ensuring that the biopsy of the tumor tissue taken for sequencing is representative;
(2) Justification of the algorithms used to select neoantigen peptides;
(3) Assessment of controls over automated parallel manufacturing processes;
(4) Establishing the consistency of a test product across multiple batches based on agreed representative quality attributes;
(5) Using pooled stability data from multiple batches.

Very recently (February 2025), the UK Medicines and Healthcare products Regulatory Agency released “Draft guidance on individualized mRNA cancer immunotherapies” [126]. Unlike the South Korean document, the draft guidance is specific to mRNA-LNP therapies but also contains guidance on neoantigen identification and selection. However, there is more specific information provided on proposed considerations around CMC/product manufacturing, non-clinical and clinical aspects for mRNA INT. The draft guidance also emphasizes the importance of having processes in place to effectively track and trace samples from the patient biopsy stage through the design, manufacture, shipping and patient administration stages to ensure that the correct product is administered.

As the technology becomes more established, it is anticipated that representative batches will be used to assess the in vitro expression of test INT mRNA products, and to confirm that products remain immunogenic (given the necessary time lag between mutation sequencing and delivery of therapy). Similarly, it is anticipated that toxicology, immune induction in vitro and delivery efficacy in vivo will utilize representative batches. Apart from the use of representative batches, we anticipate that another potentially useful approach will be the provision of regulatory data on products manufactured within certain bounds of total mRNA length and structure, and the assessment of the expression of sequences that encompass the extremes of potential INT products in the consideration of various CMC topics such as product specifications and shelf life. Regulators will also be required to adapt their manufacturing oversight to review facilities that conduct simultaneous, very small-scale manufacturing of a product under GMP conditions.

Finally, both rare disease and INT mRNA therapies are low-volume individual products and will require the development of efficient distribution protocols (potentially different for those used for large-scale vaccine distribution during the SARS-CoV-2 pandemic) so that they can reach affected patients in as timely a manner as possible. Customs and importation protocols will also need to be refined to avoid access delays, for example, whether individual import licenses would be required for each INT product or whether they could also be considered a single product at the border. Import and distribution chains will also require alignment when two agents are involved, such as a checkpoint inhibitor (which can be stocked in bulk) and the patient-specific mRNA INT.

## 6. Emerging Regulatory Trends and Issues

### 6.1. Need for Greater International Alignment in Regulatory Pathways

A significant challenge for the commercialization of mRNA products is the lack of consistency in how different mRNA products are currently classified in a regulatory sense, both within and between regulatory agencies [100,127]. This can lead to differences in data requirements and regulatory submission dossier structure for different mRNA products even though they may be developed in the same facility using the same technology and have very similar structure and composition. There are also consequences that flow from regulator’s willingness to assess data in a platform manner or otherwise strictly on a product-by-product basis. In the absence of assessment of data using a platform approach, different evaluators within the one regulatory agency could potentially require different specifications or reach different shelf life recommendations for products with very similar structure and composition. These differences in regulatory procedure also have implications as it can require a substantial effort to restructure a regulatory dossier from a medicines structure to a biologicals structure, so submission and therefore regulatory review and potential patient access are delayed.

The description of mRNA products, which do not alter the recipient’s genome, as “gene therapy” could lead to increased vaccine hesitancy among the public as it would be unrealistic to expect the patient to differentiate between gene therapies such as viral vector or DNA therapies and mRNA products. In the US FDA, mRNA products are regulated as biologicals by the CBER (Center for Biologics Evaluation and Research). Within the CBER, vaccines are reviewed by the Office of Vaccine Review and Research, while for therapeutics, the mRNA platform is regulated by the OTP (Office of Therapeutic Products). There is still uncertainty regarding the classification of mRNA therapeutics by the FDA. Overall, OTP has classified mRNA therapy as “gene therapy”; however, there is some variation in how the FDA has applied gene therapy guidance requirements. This is important as the requirements for gene therapy products can be very different. Gene therapy products are typically excluded from ICH guideline S9, which requires a leaner preclinical package, and instead are likely subject to ICH guideline S12. ICH guideline S12 requires full preclinical studies in US FDA Investigational New Drug (IND) submissions. These may include biodistribution and genotoxicity studies, safety pharmacology studies, long-term carcinogenicity studies and longer-term toxicology studies [128].

The potential consequences of mRNA being categorized as gene therapy could be very significant. For example, in recent US FDA draft guidance [129], the agency recommends long-term safety monitoring for up to 15 years of follow-up for gene therapy products, to monitor potential delayed adverse events such as cancer or off-target effects, even though this is much more plausible with products such as viral vector therapies. In the EU, the classification is even more complex. mRNA vaccines against infectious diseases are reviewed as vaccines by the EMA as “immunological medicinal products”. In contrast, mRNA therapeutics are reviewed by the EMA as gene therapy medicinal products, and a type of Advanced Therapy Medicinal Product (ATMP). A new definition of Gene Therapy Medicinal Products (GTMPs) has been proposed by the European Commission (EC), subsequently amended by a draft of the European Parliament (EP). The revised draft definition provides a clear distinction between products that edit the host genome and those that do not, a differentiation agreed upon by both the EC and the EP. Additionally, the category of nucleic acid has been expanded to include both synthetic and biological origins [128].

The US FDA and EMA also apply differences to the definition of mRNA/mRNA-LNP drug substance versus drug product, which creates further complexity in meeting regulatory data requirements during manufacturing and product analysis. There are also differences between the US FDA and EMA in how the lipids within the LNPs are treated, with differing classification as excipients or starting materials, affecting the level of safety and characterization data that are required to be submitted [130].

In some other countries such as Australia, both mRNA vaccines and therapeutics are regulated as medicines, rather than as biologicals. In Switzerland, products with defined nucleic acid sequences (including mRNA) are considered biologically active genetic material and regulated in a similar way to gene therapy products [131].

A final area of limited regulatory alignment between countries relates to the requirement in some countries for separate approvals (from a separate regulator) to manufacture and/or utilize particular mRNA products in clinical trials or for commercial use which are deemed to be, or contain, genetically modified organisms (GMOs). For example, in Australia, while no approvals are required from the Australian Gene Technology Regulator for patient administration of conventional mRNA products, the initial manufacturing step in which plasmid DNA is expanded by fermentation in an *E. coli* host would be seen as a GMO step with the non-conjugative strains of *E. coli* containing the plasmid as the GMO and thus require a license from the regulator. However, for other mRNA vaccines and therapeutics, such as self-amplifying mRNA or ex vivo mRNA/CAR-T therapies, GMO licenses are also required for clinical trials.

### 6.2. Differing International Approaches to the Regulatory Application of Platform Technology

The WHO and several leading regulators have identified the development and regulatory advantages of treating mRNA as a platform technology, although some other regulators have not at this stage announced plans to develop general platform guidance and have utilized the approach that “the platform concept can be treated as prior knowledge which is similar to any medicinal product development” [131,132].

Platform technology is most applicable to the Quality/CMC part of mRNA-LNP product development, although there are significant implications for non-clinical and clinical development too. Platforms can comprise the following:

(1) Starting materials: including DNA plasmids, enzymes, cell banks for expression systems;
(2) mRNAs that encode antigens of interest;
(3) mRNA-LNP control and testing of LNP size and mRNA encapsulation;
(4) Unit operations throughout the manufacturing process;
(5) Analytical techniques throughout the manufacturing process—identity, quantity, purity, integrity, characterization, potency and safety (contamination);
(6) Approaches to the validation of processes and methods used for manufacture and analysis;
(7) Understanding of the degradation pathways and metabolism of mRNA and LNP components in consideration of non-clinical assessment and determination of shelf life;
(8) Clinical experience justifying specification limits for certain shared attributes between products, such as particle size and product-related impurities.

As outlined in our earlier review [1], non-clinical development of mRNA products can also extensively utilize the platform approach in dose-finding, pharmacokinetic, biodistribution and toxicological studies. More specific to the individual product is the rationale for antigen selection/epitope choice and studies on the extent and duration of immune response or persistence of the replaced protein in vivo.

There are several vaccine and therapeutic development strategies that can be considered as platforms and thus could utilize platform technology. Apart from mRNA vaccines and therapeutics, these include protein subunit and virus-like particle vaccines, viral vector vaccines and therapeutics, cell and gene therapies such as chimeric antigen receptor T-cell (CAR-T) therapies as well as monoclonal antibody and biosimilar products.

At this point, regulators internationally seem to be considering three broad pathways for the evaluation of mRNA products. These can loosely be categorized as relying only on “prior knowledge”, non-technology-specific platform approaches and mRNA platform-specific approaches (potentially contemplating the development of platform technology master files). The long-established concept of regulatory prior knowledge is, for example, described as a platform approach in several ICH (International Conference on Harmonisation) guidelines. In the ICH Q11 guideline [133], platform manufacturing is defined as “the approach of developing a production strategy for a new drug starting from manufacturing processes similar to those used by the same applicant to manufacture other drugs of the same type”, while in the ICH Q14 guideline [134], a platform analytical procedure is defined as “a multi-product method suitable to test quality attributes of different products without significant change to its operational conditions, system suitability and reporting structure … would apply to molecules that are sufficiently alike with respect to the attributes that the platform method is intended to measure”.

While the prior knowledge approach embraces elements of platform technology, it has its deficiencies. Without specific guidance, there is a strong likelihood of evaluator-to-evaluator variability within an individual regulatory agency in how they utilize prior knowledge in product reviews, and there is no clarity for product developers on platform aspects. Thus, the prior knowledge approach may not lead to development or regulatory efficiencies nor a systematic utilization of learnings from the platform. The second group of platform technology approaches are “non-technology-specific”. Examples from the EMA and US FDA follow. The EMA guidance on biotechnology substances platform manufacturing [135] provides information for developers to support “a production strategy for a new drug starting from manufacturing processes similar to those used by the same applicant to manufacture other drugs of the same type …”.

In early 2024, the US FDA undertook a public consultation on draft regulatory guidance on a proposed designation program for platform technologies [136]. Section 2503 of the US Omnibus Appropriations Act of 2023 required the FDA to establish a program for the designation of platform technologies, so that sponsors may “reference or rely upon data and information” from a previous application for a drug or biological product that incorporates or uses the same platform technology. mRNA products were one example of the types of products in scope. The platform designation process aims to allow sponsors to “manufacture more than one product through a standardized process and provide predictability on product regulatory review”, by enabling information from a prior product to be re-used in a subsequent application, including consideration of prior GMP inspection findings. Potential benefits from receiving a platform designation include early and continuous interactions by the developer with the FDA and potentially an expedited review. In order for platform technology to be used to its fullest potential, it would be best for designation to commence early, and for each of the CMC, non-clinical and clinical uses of platform data to be in scope. As the extent of prior knowledge around mRNA products is already quite extensive, a major consideration is how that information will be provided by product developers to regulators and maintained in a coherent fashion. The FDA’s draft approach provides significant flexibility but lacks clarity on how the prior knowledge will be maintained.

The final group of platform technology regulatory guidance and approaches under consideration by regulatory agencies and related bodies are mRNA product-specific. In September 2022, the WHO Expert Committee on Biological Standardization developed platform technology guidance for prophylactic mRNA vaccines [137], while in 2023, the EMA consulted on the development of a platform guideline on quality aspects of mRNA vaccines [138]. The EU Directorate for Quality of Medicines and Healthcare commenced a consultation on the production and control of mRNA vaccines in May 2024, and in January 2025, they announced that in July 2025, three new texts will be published in the European Pharmacopeia covering mRNA-LNP vaccines for human use, mRNA active substances used for vaccine manufacture and linear DNA templates used as starting materials [139].

### 6.3. mRNA Platform Master Files

To establish an efficient process for developing a regulatory dossier for a new mRNA product within a platform or supporting the evaluation of changes to a product as part of the mRNA platform, a sponsor could create a master comparability protocol for that product. The master comparability protocol would include specific tests, analytical procedures, and acceptance criteria for all specified changes expected over the lifecycle of the mRNA platform product. Boundaries for acceptance criteria can be based on process knowledge, with different products using the same LNP (or potentially different LNPs with enough process knowledge), as well as the clinical experience generated with pre-licensure materials encompassing a range of values for the critical quality attributes.

Where changes are more significant in that there is a very different mRNA sequence targeting a new indication, comparability assessments could still streamline product development and regulatory review, and the concept of a master comparability protocol could be suitably adapted. In the case of a new mRNA sequence based on an established mRNA platform, the only manufacturing-related change is the manufacture of the new DNA plasmid that is then used to manufacture the corresponding mRNA sequence. This portion of the manufacturing process could be described in a comparability assessment that would reference the drug or active substance master file (DMF) for the mRNA platform manufacturing equipment, flow and process, among other things. This assumes all mRNA-LNP products referencing this DMF are formulated with the same LNP and buffer components and excipients, and use the same equipment and process.

The master comparability protocol, together with other product- and sponsor-specific information on the product, particularly CMC information, could be included in an “mRNA platform master file”. This platform master file would be a company-specific commercial-in-confidence document submitted to a regulator, which would complement but not duplicate the development of public regulatory guidance which relates to mRNA platforms, rather than specific product families.

An important regulatory concept that is in use in some countries such as the US or Canada is the master file (MF). One MF covering a specific drug component can be used across several products that use the same component, alleviating the need to submit and review the same information repeatedly across products. In the case of mRNA products, the information on the lipid mixture used to produce the nanoparticle can be included in a separate master file that can be cross-referenced in any product file that contains it. This concept could be generalized to cover one or several platform technology building blocks such as the LNP itself.

The EMA has also implemented veterinary vaccine platform technology master files (vPTMF) since January 2022 [140], and while these are for veterinary vaccines, it is reasonable to expect they may guide future thinking at the EMA on human platform technology products. The guidance describes a platform technology master file as “a file that contains all data relative to the platform for which there is reasonable scientific certainty that they will remain unchanged regardless of the antigen(s)/gene(s) of interest added to the platform” which “aims to avoid the unnecessary re-submission and re-evaluation of data relating to a vaccine platform technology used in an authorized IVMP (investigational veterinary medicinal product) for the authorization of subsequent vaccines”.

There are several analogies between this concept and the use of drug or active substance master files (DMFs or ASMFs) in regulatory submissions. DMFs have particularly been used to support regulatory submissions around the drug substances for generic medicines, although they are used more widely for a wider range of applications to enable the provision of confidential product information to regulators to support the applying sponsor’s application, without disclosing proprietary information. DMFs can provide detailed information about processes and facilities used in manufacturing as well as packaging and storage. They can also in some cases include preclinical information such as toxicology and clinical risk evaluation and mitigation strategy information.

It is proposed that an analogous approach to the development and use of mRNA platform master files be explored. While the intent of these master files could also be to protect confidential information for products that are licensed from an mRNA product manufacturer to a separate sponsor, the overarching purpose in most cases is to enable the development of and sharing with regulatory agencies of a single set of manufacturing and analytical processes for a proprietary family of mRNA products.

### 6.4. mRNA Vaccine Laboratory Lot or Batch Release

In several countries, vaccines are required to undergo individual lot or “batch” laboratory testing—depending on the local legislation, testing is conducted either by the manufacturer, the regulatory agency or a third-party laboratory network, e.g., the Official Control Authority Batch Release (OCABR) laboratory in Europe [141]. Lot release has been considered necessary because of concerns that there is a risk of greater batch to batch variation between manufacturing batches of vaccines than other biologics and pharmaceuticals because of the greater molecular complexity of biological medicines than chemically manufactured medicines. For this reason, regulators require testing of the final product for potency assurance (e.g., concentration and functionality of the active ingredient) and safety (typically of contaminants that could cause adverse reactions). The full set of analytical tests used for vaccine QA/QC is shared with the relevant regulatory agency and lot release specifications and characterization tests will require agreement with them as part of the regulatory market authorization process.

The EU has developed specific protocols for each type of vaccine for different antigenic targets [142] with some differences for different antigens across a given vaccine platform. They have recently updated their protocol for COVID-19 mRNA vaccines, with a new version in force from January 2024, but a general mRNA vaccine protocol is needed.

Many of the same platform considerations that have been applied to analytical testing to support CMC controls for mRNA vaccines should also be able to be applied to batch release considerations, and it will be important to specify this in future regulatory guidances. For example, the EU OCABR guidelines for COVID-19 mRNA vaccines specify that on at least 15 containers of each final lot, official control laboratories should, apart from requiring documentation on production volumes, storage temperatures and time and expiry dates for intermediates and final product, test for appearance, identity (for each antigen/strain, if it is a multi-strain vaccine) and integrity.

However, there is no international alignment on the processes or requirements for lot testing, and this can have implications for sponsors of imported mRNA vaccines. Further, the rationale for lot testing being required for vaccines derived from the origin of earlier vaccines as inactivated or killed cellular biological products. Given that mRNA vaccines are derived from in vitro translation of DNA, it could be questioned whether lot release testing should be required at all for mRNA vaccine products. Alternate approaches to manage safety and quality risks throughout the lifecycle could be considered for mRNA products, including some of the approaches proposed for individual neoantigen therapies in this review.

### 6.5. Critical Requirements for International Regulatory Alignment

With the large number of products in late-stage clinical trials, it is encouraging that different regulatory agencies and related organizations have developed or are developing regulatory guidance for mRNA vaccines and related mRNA products. It is noteworthy that in preparation for regulatory review of individualized neoantigen therapies for various oncology indications, the Korean and UK regulators have developed specific guidance documents (see Section 5.5). However, there are unfortunate inconsistencies in the regulatory treatment of mRNA products in different jurisdictions, which will only delay access to new products globally. Some of the inconsistencies that need to be addressed include the following:

- The need to exclude all conventional (non-self-amplifying) mRNA-LNP products from the definition of “gene therapy medicinal product” in the EU [127]. The definition should not encompass products that edit or alter the human genome;
- Agreement on a wider, more flexible definition of platform technologies;
- Prompt publication of detailed regulatory guidance on platform technology requirements, especially relating to chemistry, manufacturing and controls;
- Establishment of platform technology master file option for human mRNA products;
- Consensus on the definition of different mRNA-LNP vaccine components as starting materials or excipients [130] and of mRNA-LNP as drug substance or drug product;
- International consensus on lot release requirements for mRNA vaccines.

While several regulatory agencies and related bodies have developed or are developing regulatory guidance, the main source of collaborative guidance around medicine and vaccine development remains the ICH (The International Council for Harmonisation of Technical Requirements for Pharmaceuticals for Human Use). However, the ICH is slow to develop new guidance documents and advocacy in other forums such as the ICMRA (International Coalition of Medicines Regulatory Authorities) may be required to progress these recommendations.

## 7. mRNA Product Safety

While critical for both vaccines and therapeutics, vaccine safety is appropriately closely scrutinized because most vaccines are administered to healthy people and to large numbers of children. While the mRNA COVID-19 vaccines were authorized for both children and adults, the majority of doses were administered to adults. Even so, initial trials were conducted with adult cohorts, and the initial regulatory approvals for the mRNA COVID-19 vaccines were for adults, before being extended to children following proper completion of clinical trials in pediatric age groups. Regulatory safety monitoring does not commence with post-market monitoring but rather is integral throughout the product development lifecycle [142]. Safety considerations are integrated into the design algorithms for many mRNA products, and toxicological studies are a major part of the preclinical development of all products, prior to any first-in-human clinical trials. As for any new product development, all clinical trial protocols closely evaluate safety, in first-in-human trials prior to the introduction of a new treatment cohort as well as prior to approval for progression to subsequent trial phases involving more subjects.

Both clinical trials and real-world experience have shown that mRNA COVID-19 vaccines have a very good safety record; indeed, they became the mainstay after rare fatalities were associated with viral vector vaccines. The good safety record has also been observed in clinical trials of mRNA vaccines for indications other than COVID-19, and the first non-COVID-19 mRNA vaccines (targeting the respiratory syncytial virus pathogen) have now been approved in a large number of countries, including in Europe and the USA [143].

### 7.1. mRNA Vaccine Reactogenicity

Reactogenicity, which is due to an inflammatory response to vaccination, is a characteristic of many vaccines and was recognized well prior to the roll out of mRNA vaccines during the COVID-19 pandemic. It has been described as encompassing “injection-site pain, redness, swelling or induration (hardening of areas under the skin due to inflammation) at the injection site as well as symptomatic symptoms, such as fever, myalgia or headache” [144]. While reactogenicity effects are largely mild, short-term and self-resolving, they can lead to fear and loss of confidence in vaccination and vaccine hesitancy. Reactogenicity is due to an immune response to vaccine components, in the case of mRNA vaccines, the production of type 1 interferon. Both the mRNA and lipid nanoparticle components may contribute [145], with the LNP components particularly responsible [146,147,148]. There is potentially a relationship between the reactogenicity of the LNP and adjuvanticity, with the ionizable components of the LNP having adjuvant and immunostimulatory activity [148]. mRNA and LNP have divergent impacts on reactogenicity [149] and it is possible that further developments in LNP composition and design could reduce reactogenicity [146].

The main reactogenicity symptoms were similar for the two main mRNA vaccines, BNT162b2 and mRNA-1273 [150]. While all vaccines can be reactogenic, the mRNA COVID-19 vaccines have been associated with higher short-term reactogenicity than other COVID-19 vaccines. A systematic review which assessed 28 clinical trials of different COVID-19 vaccines as boosters [151] found that mRNA vaccines were the most reactogenic, compared to viral vector and protein subunit vaccines, and inactivated vaccines were the least reactogenic. Other than the ChAdOx viral vector vaccine early in the pandemic, the use of non-mRNA vaccines has been much more limited, however. In reports to the US CDC v-safe surveillance system, a smartphone-based active surveillance system in the US, about half to two-thirds of those who made reports reported reactogenicity, with higher proportions in females and those under 45 years. Local reactions were more common than systemic reactions, but new or worsening reactions were uncommon 2 weeks after vaccination, with fewer than 3% of people reporting any reactions on day 14 [152]. It is important to note that there is a general correlation between efficacy and reactogenicity, emphasizing the importance of assessing the overall benefit–risk profile of a vaccine rather than solely its reactogenicity profile.

### 7.2. Adverse Events of Special Interest

More serious adverse events often termed “adverse events of special interest (AESIs)”, are quite rare but not unknown. A range of rare, serious adverse events were reported at low frequencies in mRNA-vaccinated adults in clinical trials, including myocardial infarction, Bell’s palsy, cerebral venous sinus thrombosis, Guillain Barre syndrome, pulmonary embolism, stroke, thrombosis with thrombocytopenia, lymphadenopathy, appendicitis, herpes zoster reactivation, neurological complications and autoimmunity as well as myocarditis and pericarditis [153], but the frequency of many of these events was not different to that observed in the placebo arms of the trials [154].

The most significant AESI that has been clearly established for mRNA COVID-19 vaccines, myocarditis and pericarditis, is rare, occurring in only 1 or 2 cases per 100,000 people, and at much lower frequencies than in populations with SARS-CoV-2 virus infection [155,156]. At such low frequencies, these adverse events may not manifest in a clinical trial with 10,000 or 20,000 subjects in the vaccinated trial arm. The highest risk of myocarditis and pericarditis was in males under 40 years of age [157,158], although a review of safety outcomes following COVID-19 vaccination in 5.1 million English children emphasized that the overall safety profile was positive [157]. A recent study proposed a mechanism for myocarditis and pericarditis resulting from COVID-19 mRNA vaccines, identifying an inflammatory reaction in the cardiac tissue [159] with increases in circulating interleukins, chemokines and matrix metalloproteases. There was no evidence that antibodies to the virus spike protein were involved.

While some have proposed that myocarditis and/or myocarditis may be a class effect of mRNA vaccines, the evidence for this is mixed. For example, no myocarditis or pericarditis safety signals have been observed in the recently approved mRNA RSV vaccine or in other mRNA vaccines under development. Evaluation of the longer-term safety and effectiveness of mRNA-1273 (Moderna Inc., Cambridge, MA, USA) in individuals enrolled in the original clinical trials [160] showed that the safety outcomes for boosters were similar to primary vaccination. One case of myocarditis was observed among over 16,000 people receiving the booster of the vaccine. There have been some deaths attributed to myocarditis following vaccination with mRNA COVID-19 vaccines [161], but overall, patients with myocarditis after COVID-19 vaccination do well, although some require medical care for several months. A recent study on the longer-term prognosis [162] using French national health data indicated that cardiovascular events after myocarditis following COVID-19 vaccination were less severe than after other causes of myocarditis.

Some studies have postulated possible mechanisms for myocarditis and pericarditis from mRNA COVID-19 vaccines, but there is no clear consensus and several confounding issues [163]. Possible mechanisms include that the immune system might detect the mRNA in the vaccine as an antigen, resulting in the activation of pro-inflammatory cascades and immunological pathways in the heart. This hypothesis is not supported by the lack of immune-related adverse effects in other organs. Molecular mimicry between the spike protein of SARS-CoV-2 and cardiac proteins is another possible mechanism. It has also been postulated that testosterone may have a role given the higher incidence of myocarditis in younger males. Additionally, Kadkhoda [164] postulated that circulating mRNA-LNPs could be endocytosed by cardiac tissue, with local production of spike proteins on cardiac cells attracting neutrophils that also express ACE2 on their surfaces. Again, the question of why this was not observed with other organs was raised. Altman et al. [165] proposed that myocarditis may be caused by inflammatory cell infiltrates or by microvascular thrombosis. Buoninfante et al. [166] have recently reviewed possible mechanisms involving spike proteins, lipid nanoparticle pro-inflammatory responses, sex hormones and autoimmune and genetic factors but concluded that “there is still no clear understanding of the biological mechanism/s responsible”.

A wide range of different types of vaccines have been associated with myocarditis and pericarditis and include live attenuated and protein subunit vaccines such as vaccines for smallpox, anthrax and possibly influenza [165,167]. In addition, an assessment of reports from the WHO VigiBase database demonstrated that the Novavax COVID-19 protein vaccine has been associated with a similar frequency of myocarditis events as the mRNA COVID-19 vaccines [168]. It is unclear, therefore, whether rare myocarditis will be observed with other mRNA vaccines or therapeutics, and if it is, whether there would be a difference in frequency compared with other vaccines developed for the same disease.

In many countries, the largest numbers of adverse events were reported for COVID-19 vaccines, including mRNA vaccines, but this should be viewed against the massive numbers of vaccinations administered for COVID-19 and the promotion by government and other health agencies for individuals to report adverse events, even if the reporting individual was unclear whether there was any potential association with vaccination. In addition, the majority of the reported adverse events were reactogenicity-related.

### 7.3. Attribution of Adverse Events

It is important to differentiate adverse events which have been reported from those that have been confirmed and attributed to vaccination including through in-depth population-based studies and statistical approaches. Studies of the background rates of particular conditions in the community, particularly assessed by age and sex, are critical [169]. The WHO has published guidance on the causality assessment of adverse events following vaccination [170]. Where there is at least a reasonable possibility that a vaccine could have caused a suspected side effect, regulatory agencies include this information in the product information document or label for the vaccine. The information is also summarized in the consumer package leaflet, e.g., by the European Medicines Agency [171,172]. Apart from myocarditis and pericarditis, both vaccines list other possible adverse events:

(1) Bell’s palsy (temporary one-sided facial drooping);
(2) Swelling of the face;
(3) Severe allergic reaction;
(4) Extensive swelling of the vaccinated limb;
(5) Swelling of the face in patients who have had facial dermatological fillers;
(6) A skin reaction (erythema multiforme);
(7) Unusual (paraesthesia) or decreased sensation in the skin (hypoaesthesia);
(8) Heavy menstrual bleeding.

Extensive swelling of the vaccinated limb and skin rashes (mechanical urticaria and chronic urticaria) are also listed as possible adverse events in the EMA package leaflet for the Moderna vaccine to the SARS-CoV-2 XBB.1.5 variant. A possible safety signal for acute disseminated encephalomyelitis (ADEM) and transverse myelitis for mRNA COVID-19 vaccines has been identified in a global study of 99 million vaccinated individuals [156], although a second study of 6.7 million people did not identify this signal [173]. In any event, the absolute risk of both forms of myelitis is low, being under two in a million doses. Regulators continually review safety signals to determine whether additional adverse events are identified.

Another claim that has been made by some groups is that the commercial mRNA COVID-19 vaccines contained large levels of contamination of DNA fragments from the plasmid used for initial transcription of the mRNA [174,175]. However, these claims have been strongly rebutted by regulators (e.g., the Australian Therapeutic Goods Administration, TGA [176]) and scientific groups [177]. In these rebuttals, it is noted that the fluorometric method used for DNA measurements in several of these studies provides significant over-estimates when RNA is present, that the samples tested were expired vaccine samples of unknown provenance and that the laboratories undertaking the testing appeared not to have formal accreditation as an analytical facility. It is also emphasized that residual DNA, even if it were present at high levels, cannot alter human DNA. Moreover, the TGA has published laboratory lot testing data for the commercial mRNA vaccines indicating that residual DNA levels are very low and meet international regulatory requirements [178].

### 7.4. Potential for Adverse Events with Therapeutic mRNA Products

While an area of significant clinical trial activity, therapeutic mRNA products such as those for cancers or rare diseases have not yet received regulatory approval. In addition, the numbers of patients enrolled in each clinical trial are typically somewhat smaller than for trials for the mRNA SARS-CoV-2 vaccines. The small size of the patient groups means that it remains unclear whether rare adverse events such as myocarditis will be associated with mRNA therapeutics.

The patient groups in these trials also are generally unwell compared with the healthy individuals in the trials of COVID-19 vaccines. Therefore, the databases on adverse events from human clinical experience with mRNA therapeutics are currently rather limited. The treatment-related adverse events that have been reported in clinical trials are unremarkable. In a phase 1/2 clinical trial of an mRNA therapeutic for propionic acidemia in children [100], no dose-limiting toxicities were observed. Treatment-emergent adverse events (TEAEs) related to the mRNA therapeutic were reported in 9/16 participants and included pyrexia, diarrhea and vomiting. Two of 16 participants reported three drug-related serious adverse effects (SAEs), including one participant with grade 3 pancreatitis and one participant with vascular device infection and injection-site reaction (both grade 2).

In the trial of an mRNA INT for melanoma [119], 157 patients were assigned to an mRNA-LNP therapeutic plus pembrolizumab combination (n = 107) or pembrolizumab monotherapy (n = 50). Most TEAE were grade 1–2, with grade ≥3 TEAE in 25% of patients in the combination group and 18% of patients in the monotherapy. The most common adverse events related to the mRNA-LNP were influenza-like symptoms and local injection-site reactions, which were generally self-limited and decreased in subsequent dosing cycles.

In a phase I trial of another mRNA INT autogenecevumeran for pancreatic cancer [121], the investigators sequentially administered atezolizumab (an anti-PD-L1 immunotherapy), the mRNA INT and a four-drug chemotherapy regimen. None of the 19 patients treated with atezolizumab in the safety-evaluable cohort had grade 3 or higher adverse events. One out of 16 patients treated with the mRNA INT in the safety-evaluable cohort had grade 3 adverse events (fever and hypertension). All 16 patients who received the mRNA INT had grade 1 TEAE (chills, fever, fatigue, diarrhea, alopecia, edema, anorexia, dyspnea), while six cases of grade 2 TEAE were observed (infusion reaction, thromboembolism, anemia, hypertension, vomiting).

However, from first principles, the safety profile of mRNA therapeutics is likely to be different from mRNA vaccines, due to the higher doses of products typically used (compared with vaccines) and the requirement for repeat doses at comparatively short (e.g., monthly) intervals. In addition, with a therapeutic, the immunogenic effects of both the mRNA and the LNP are generally undesirable rather than a desirable (adjuvant) effect with a vaccine. In developing these products, impacts on innate immune activation and immune tolerance should be closely examined. Immune tolerance to the protein product of an mRNA administered repeatedly could potentially lead to a loss of efficacy. However, animal studies have not shown decreases in protein expression after chronic dosing, although some studies detected antibodies to these protein products [112]. Some animal studies have also shown some liver toxicity from repeatedly administered mRNA-LNP [118].

## 8. Social License to Operate for mRNA Products

The COVID-19 vaccines have been estimated as saving almost 20 million lives in their first year of administration [179]. However, their introduction and roll-out, particularly of the mRNA vaccines, also was accompanied by concerns in some parts of society. Surveys across many countries have revealed very significant differences in consumer preparedness to take new mRNA vaccines following their regulatory approval [180].

Some expressed concerns that the mRNA vaccines had been developed and approved by regulators quickly and with little supporting evidence, and there is a wider lack of trust in the science [181]. In countries such as the US, where emergency use authorizations rather than regulatory approvals were employed, this may have contributed to this view. In Australia and some other countries, the vaccines initially received provisional or conditional approvals, and the fact that these were “provisional” rather than full approvals also contributed to the same sentiments. Significant education and communication campaigns, including from the International Coalition of Medicines Regulatory Authorities, attempted to address these issues [182].

Concerns around safety risks also contributed to vaccine hesitancy. Regulators globally published regular safety reports, but there was conflation by some sources of the reporting of adverse events with their actual causality. A recent French survey [183] identified that 62% of those surveyed felt that there was “still a lot that we don’t know about the side effects of mRNA vaccines”, and the same percentage either agreed or did not know whether “mRNA vaccines modified the DNA of those vaccinated”. Mandatory vaccination requirements at the height of the pandemic in some countries for certain occupations were also divisive, with many seeing them as necessary to limit exposure to infection in vulnerable and high-risk groups [184], while others took the view that they were a violation of human rights.

Concerns about international equity, which included limited access to mRNA vaccines by certain developing countries in first 12–18 months of vaccine availability, obstacles to the use of the technologies in these countries such as cold chain access and the lack of local manufacturing capacity were also factors in opposition to mRNA and other COVID-19 vaccines. The WHO and the Medicines Patent Pool have implemented a global mRNA technology transfer program [185], establishing a global collaborative network with a hub in South Africa. Several Asian developing and middle-income countries are also establishing a capacity for mRNA vaccine manufacture.

Vaccine hesitancy has been defined as “a state of indecisiveness regarding a vaccine decision” [186] rather than opposition to vaccination. An increase in hesitancy commenced well before the introduction of mRNA vaccines or the COVID-19 pandemic vaccines and has been considered by the WHO as a top 10 global health threat following an increase in measles cases globally. There is some debate around the best approaches to reduce vaccine hesitancy. While most agree that it is important to respect that the decision whether to be vaccinated is an individual one, some argue that government can play a stronger facilitating role by facilitating low-cost or free access in communities, developing culturally appropriate resources or investing in health systems, especially those targeting marginalized groups [187]. There is evidence that hesitancy contributed to the severity of COVID-19 waves in some countries [188,189]. Over the last couple of years, the issue seems to be “vaccine fatigue” (a loss of interest in receiving an mRNA (or any) COVID-19 vaccine), despite strong recommendations from health authorities that particularly the elderly and vulnerable should avail themselves of COVID-19 boosters. Several factors may be responsible, including evidence that the more recent variants have lower mortality and morbidity than earlier ones as well as the general desire to put COVID-19 “in the rear vision mirror”.

The final issue relates to misinformation about the safety or other effects of mRNA vaccines [190]. There has been extensive information circulated, particularly through social media, for example, that spike proteins and protein fragments encoded by the vaccines and LNPs are toxic, that mRNA or DNA copies of mRNA vaccines can somehow incorporate into the genomes of recipients of the vaccines and that mRNA vaccination has led to significant increases in death rates in the population, including celebrities who have died suddenly. Other misinformation campaigns relate to mRNA vaccines being seen as a tool of control by governments or big pharma. Even more than four years after the first mRNA COVID-19 vaccinations, misinformation is rife. A recent survey of nearly 1500 US adults published in July 2024 [191] revealed that 28% of Americans believe that COVID-19 vaccines have been responsible for thousands of deaths, while 22% believe that it is safer to contract a COVID-19 infection than be vaccinated.

Several approaches have been proposed to increase confidence in COVID-19 mRNA vaccination. There is a role for genuine community engagement and empowerment to combat mis- and disinformation. The use of community organizations and leaders who have established trust have been successful [192]. Employers and healthcare providers and local governments that had strong community connections were also seen as trusted sources of vaccination advice [192,193,194]. Traditional communication strategies from governments worldwide were often “one way”, and unresponsive to emerging community concerns; they missed opportunities to address misinformation early and insufficiently utilized social media platforms, despite this being the platform used to seek information by many in the community. Van der Linden [195] has made some valuable suggestions on how misinformation can be better managed.

The WHO Science Council has recently recommended that the WHO utilize its reputation to address misinformation and disinformation around mRNA vaccines (although many of those who do not trust government also particularly distrust international institutions). Healthcare professionals, trusted community leaders and trusted friends and family may have a greater impact. Another approach may be “prebunking”, wherein organizations pre-emptively build resilience against anticipated exposure to misinformation, including building consumer capacity to detect information manipulation and the deliberate dissemination of false information [196].

The concept of the pharma industry, in particular sponsors of mRNA vaccines requiring a “social license to operate”, will almost certainly gain momentum in the coming years [197]. The term “Social license to operate” generally refers to the ongoing approval or acceptance of an activity by the general public. The relevant activity in this case is the administration of mRNA vaccines. A social license is not a formal arrangement or contract between the parties involved but rather the use of the term implies that there is community acceptance based on a belief that the activity is necessary under the circumstances.

**Figure 2:**
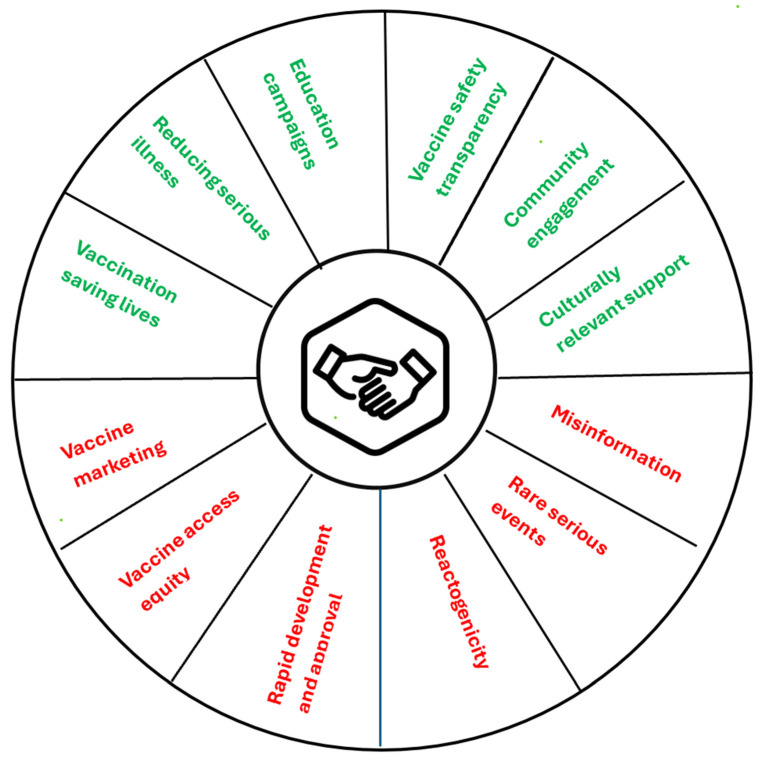
Factors influencing the “Social license to operate” for mRNA vaccines.

In Figure 2, I summarize a range of factors (shown in red text) that can negatively impact the social license to operate. These include concerns about the commercial promotion of vaccines in some countries, equity in access to vaccines by those in low-and-middle income countries, the rapidity of vaccine development and approval, vaccine reactogenicity and rare but serious adverse events and the impact of misinformation campaigns, in which inaccurate information on issues such as vaccine safety is communicated, usually through social media but also by elected officials. Actions that can improve the social license to operate for mRNA vaccines and therapeutics are shown in green, and include education campaigns, particularly those providing evidence that mRNA vaccination saves lives and can prevent serious illness, transparency by regulatory agencies, government and industry around vaccine safety, and the design and delivery of community engagement programs, including in a culturally relevant way for minorities.

The “social license” term was initially coined in the 1990s by the forestry industry and was related to the environmental “license” that they could achieve with society by demonstrating that their harvesting activities were responsible and environmentally sustainable [198]. It has since been used more widely by the mining industry [199] as well as in aquaculture but is increasingly being applied to banking, public policy and tourism; in health, it is coming to the fore in the discussions on acceptable boundaries for health research and the use of artificial intelligence and other approaches to mine sensitive personal health data [200]. Some jurisdictions, such as the USA and New Zealand, permit direct communication on vaccines between companies and consumers, while many OECD countries do not. While some studies have suggested that vaccine sales increased following direct to consumer advertising [201], other studies have shown that vaccine promotion requires greater nuance to encourage vaccine uptake [202]. Interestingly, the social license argument has been turned on its head to emphasize the social responsibility of parents deciding to have their children vaccinated, given the implications, if unvaccinated, of the spread of vaccine-preventable diseases to others [203].

## 9. Conclusions

mRNA vaccines have been among the most widely used therapeutic products globally in the last decade. Just under 2 billion doses of the two major mRNA COVID-19 vaccines (from Pfizer-BioNTech and Moderna) have been administered in the EU, US, Canada and Japan combined, in the period to 10 August 2024 [33]. A wide range of mRNA products for infectious disease as well as rare disease and oncology were under preclinical or clinical development before the COVID-19 pandemic. However, from the first global authorization of a COVID-19 mRNA vaccine, it took about 3 ½ years until the first mRNA vaccine for another disease (Moderna’s RSV vaccine) to receive regulatory approval. In contrast, based on the robust pipeline of late-stage clinical trials [1], a significant number of regulatory submissions for mRNA products is anticipated in late 2025 and the following years for a wide range of indications.

mRNA COVID-19 vaccines were rapidly developed and regulatory review and approvals undertaken efficiently without compromising product safety, quality or efficacy. Outside pandemic conditions, it will be important to maintain development and regulatory efficiencies by finalizing the development of the range of mRNA platform technology regulatory guidelines that are currently under development and to ensure that there is international alignment between regulatory approaches to the extent possible. With several mRNA therapeutics in advanced development, it is critical to obtain clarity on the regulatory approaches to be used for the evaluation of these products, in particular for individualized neoantigen oncology therapies, where the mRNA sequence in the drug product administered to each patient differs.

It is often said that “vaccination, not vaccines, prevent disease”. Therefore, incentives and barriers to vaccination are an important part of the lifecycle considerations for mRNA products. While recognizing the right of individual choice, we believe that for those facing a diagnosis of cancer or who are parents of a child with a serious rare metabolic disease, the considerations may be different. There will likely be a higher acceptance of mRNA therapeutics than for many healthy individuals considering vaccination against a disease that they may consider they only have a limited chance of contracting.

## Data Availability

No new data were created or analyzed in this study. Data sharing is not applicable to this article.
